# Equity + Wellness: A Call for More Inclusive Physician Wellness Efforts

**DOI:** 10.15694/mep.2021.000099.1

**Published:** 2021-04-26

**Authors:** Sylk Sotto-Santiago, Dianne Ansari-Winn, Chemen Neal, Michael Ober

**Affiliations:** 1 Indiana University School of Medicine; 2 The Physician Coaching Academy

**Keywords:** wellness, burnout, equity, diversity, inclusion, belonging, coaching

## Abstract

This article was migrated. The article was marked as recommended.

The challenges, importance, and state of physician wellness and burnout have been well documented throughout the literature.Research continues to prove the value of tools and interventions while institutions appear to be listening and adapting important practices. However, although the wellness literature encourages a review of organizational challenges, local needs, and individual solutions, organizations may fail to align these efforts along with equity, diversity, inclusion, and belonging (EDIB). A pandemic and recent events in our society heightened awareness about health inequities, structural violence and racism, and demand that we look within our institutions and health systems. It also demands that we speak of wellness and equity together. We cannot engage in conversations about wellness without asking about equity...because equity and inclusion lead to wellness. We simply cannot expect our healthcare workforce, faculty, and physicians of color to be “well” if they are experiencing exclusion and inequality. In this article, we present the concepts of *inclusive excellence* and *leading with wellness in mind* while calling for more inclusive physician wellness efforts.

## Introduction

The challenges, importance, and state of physician wellness and burnout have been well documented throughout the literature ( West *et al.,* 2016; Shanafelt *et al.,* 2015; West, Dyrbye, and Shanafelt, 2018; Shanafelt, Dyrbye, and West, 2017). Research continues to prove the value of tools and interventions while institutions appear to be listening. Organizations are investing in wellness and elevating the issues well into the C-suite, with the creation of Chief Wellness and Happiness Officers ( Kishore *et al.,* 2018; Butcher, 2019; Williams, Berger, and McClendon, 2005). Academic medical and health systems are offering everything from commensality opportunities, learning communities, coaching, and tools that build resiliency, self-awareness, and mindfulness. The unquestioned subscription is that wellness brings value and it is something we should all care about. However, although the wellness literature encourages a review of organizational challenges, local needs, and individual solutions, organizations may fail to align these efforts along with equity, diversity, inclusion, and belonging (EDIB). For this perspective, we adapt and define physician wellness as quality of life, including the presence of positive mental, social, and integrated well-being experiences **in connection with inclusive excellent environments** that allow physicians to develop their full potential across personal and work-life domains ( Lenoski, 2019).

The literature has centered on multifactorial issues that are often out of a physician’s immediate control, greatly highlighting inefficiencies and infrastructure limitations, and the importance of organizational development.There is also an emphasis on individual growth focused on resiliency ( APA, 2020). Resiliency, the process of adapting well in the face of adversity, trauma, threats, and stress, can lead to an incomplete approach as in its individualistic style ignores systemic issues, adds significant personal burden and does not place individual experiences in relation to society. Individual resilience and institutional resilience are both needed.

That society is shaped by pervasive structural racism. In academic medicine, structural racism dominates organizational systems of privilege and bias that disadvantage not only minoritized physicians, but minoritized patients, learners, trainees, and team members ( Chesler, Young, and Beale, 2013; Eagan and Garvey, 2015). As proponents of equity and inclusive excellence, one must ask how wellness needs differ within diverse provider and learner populations, and whether we are meeting the needs of all members of our workforce.

Simply put, inclusive excellence means embedding EDIB in everything the department does. It also means championing “equity-mindedness” -a mode of thinking exhibited by individuals who call attention to patterns of inequity ( AAC&U, 2005; Williams, 2007). It is the recognition that the department’s success is dependent on how we value, engage, and include the rich diversity of faculty, students, trainees, and staff ( Sotto-Santiago, 2020; Sotto-Santiago, Ober, and Geraci, 2020). Leading with wellness in mind should also inform decision-making.

A pandemic and recent events in our society heightened awareness about health inequities, structural violence and racism, and demand that we speak of wellness and equity together. Wellness and equity are not independent from each other. Not long ago, we talked about the experiences of faculty and physicians of color in medicine and wondered: how can we talk about wellness without equity? ( Ansari-Winn and Sotto-Santiago, 2018). We know that these disparate experiences exist, why aren’t we talking about a connection with wellness in ways that also move the equity needle? ( Chesler, Young, and Beale, 2013; Steele and Aronson, 1995; Sue *et al.,* 2007; Sue *et al.,* 2019; Turner, Gonzalez, and Wood, 2008; Hartlep and Ball, 2019).

Racism has deep effects on the health and well-being of individuals. Numerous studies have shared the persistent disparities in key health indicators such as access, resources, and outcomes amongst groups across the U.S. ( Baciu *et al,* 2017). A 2019 AAMC report on Burnout Among U.S. Medical School Faculty highlights women faculty having higher levels of burnout than men, a 14 point difference (35%). The report also highlights historically marginalized/minoritized faculty at 9 points higher than the academic medicine majority (35%) ( AAMC, 2019). Although, underrepresented physicians appear to be less likely to report burnout,it does beg the question as to why and if navigating such spaces in itself is a sign of highly developed resilience.

This is not a critique of wellness efforts; we need them, but simply the exhortation to not have wellness conversations without also speaking about equity and inclusion. We need to talk about decisions in ways that ask how they will differently impact diverse groups. We need to remind our leaders that we have a responsibility for everyone in the system. **There cannot be conversations about wellness without asking about equity...because equity and inclusion lead to wellness.** We simply cannot expect people to be “well” if they are experiencing exclusion and inequality. Extant literature suggests that the organization plays a critical role in whether faculty physicians remain engaged or burned out ( Garcia *et al.,* 2020; Gazelle, Liebschutz, and Riess, 2015; Shanafelt and Noseworthy, 2017). Strategic frameworks do not have to be complex. In what follows, we propose a series of questions within the framework of the *Wheel of Life.*

## Equity + Wellness

The Wheel of Life is a popular tool, used in coaching and other areas to assess life fulfillment.In coaching, the goal is to self-reflect while providing a visual representation of balance and fulfillment. Through this self-reflection, individuals can celebrate and enhance areas in which they see strength and focus on those that need further development. Based on seven aspects of wellness: career, financial, spiritual, physical, intellectual, family, and social needs, we pose the following questions for consideration as an example that can be adapted by institutions’ wellness leads.

**Career** wellness has been defined as engaging in work that provides personal satisfaction and is consistent with the individual’s values and goals ( Buffington, Melnyk, and Neal, 2018). This type of reflection invites individuals to look at the organizations’ EDIJ record. Do individuals feel like they work for an organization that has their best interests in mind? What do employee satisfaction and engagement surveys tell us about minoritized faculty perceptions of the organization? Does the organization consider the unique contribution that minoritized physicians make that are not often included in advancement decisions? Does the organization recognize the signs of minoritized faculty burnout and under what circumstances have they experienced more of these symptoms? What are the unspoken and unwritten expectations that we hold for minoritized physicians? What is the attrition rate of minoritized physicians and how this attrition is connected to hostile climates or the misalignment of individual values with those that the organization professes and does not realize?

**Financial** decisions are not exempt from institutional bias. This institutional bias operates in ways that result in the advantage of certain groups over others through systemic policies, procedures, and guidelines. Institutional bias can affect physician financial health. A recent study determined that patient satisfaction surveys as a metric for quality-based financial incentives carry a risk of bias toward women and underrepresented physicians, and may perpetuate pay inequities in financial incentives ( Sotto-Santiago, Slaven and Rohr-Kirchgraber, 2019; Chen *et al.,* 2020). It is important to ask if “good intention” initiatives have been assessed for institutional bias? Has the leader considered all factors impacting one’s ability to obtain an incentive, such as race/ethnicity or gender? What do fair compensation and reimbursement look like? Has the organization changed the way that they reimburse over time in favor of some groups?

**Physical** wellness has been of great focus in wellness initiatives everywhere from gym memberships to chair yoga, but are all levels of physical abilities respected and embraced? Do physical wellness programs address the need of those with different abilities? Are they able to actualize their health goals in terms of healthy eating habits, sleep, and exercise? Do they have medical or mental health needs or challenges? How can they mitigate the effects of stress? **Spiritual** and **Family** wellness should also question if all spiritual beliefs/faiths are acknowledged and respected? What is an ideal description of their spirituality, values, and family structure? Are diverse forms of families embraced and respected? Are our policies employee-family friendly across all different types of families? How can they develop their spirituality or have better relationships with family/loved ones? Are there aging parents, or children with special needs? What about child care and work-life harmony?

**Intellectual** wellness refers to engagement in scholastic, cultural, and community activities ( Geary, 2014). Intellectual wellness is one of the key aspects of academia and healthcare systems. How do organizations stimulate personal and professional growth is critical to employee satisfaction and engagement? Are wellness initiatives and topics diverse? Are wellness presenters-speakers diverse? How is their professional development supported in terms of continued learning, opportunities to contribute academically, serving on committees, leadership development? Do all members of the workforce have the tools necessary to meet their full potential or are opportunities reserved for members of the majority? If they pursue topics such as healthcare disparities or providing care for under-resourced populations, are they supported in this work?

**Social** wellness speaks to how healthy relationships help you navigate this world ( NIH, 2018). Social wellness involves building healthy and supportive relationships while fostering genuine connections. As we help build these connections within our organizations, we should evaluate: what kind of affinity communities do we have in place that support social wellness? Are we helping individuals identify who is in their network of support and how to build it? Are they experiencing racism, discrimination, explicit or implicit bias at work? How are existing social connections meeting their needs? How is the environment affecting their wellness? When physicians are supported in their vision, then both the physician and the organization benefit.

## Implications

The practical implications of this call are not limited to strategic decisions. Individuals can also see in this model a framework from which to compare their values and strengths with their organization. If the organization has created a wellness program that is truly supportive to the minoritized physician, this collaboration can no doubt be beneficial to both parties. We want to emphasize it is the onus of the organization to create a space where minoritized physicians feel safe to share their insights; and not the responsibility of the physician to try to carve out a niche in an organization that is not attuned to them. This type of *wellness-with-equity-in mind* allows us to realize increased physician longevity.

There are several opportunities to expand on this work. First, wellness and equity programming must be evaluated in order to weigh success. Simply, imitating programs may have contributed to the unquestioned subscription to a “one-size fit all approach” in wellness. Even when wellness scholars call for inclusion in wellness efforts, the problem is that repeatedly, leaders fail to consider the unique institutional climates that minoritized faculty physicians are navigating. Secondly, if individual resiliency programming is a must, then so is institutional resilience. Future work should address the institution’s ability to cope with and ultimately grow from environmental stressors. Institutions react in many ways to external factors and unfortunately, in their path to searching for solutions they transfer undue burdens. We do not have to go very far to find examples of this. It is currently happening in our society and medicine; due to clear structural violence as exemplified in the death of Black men and women, and the clear structural racism embedded in our health care systems exemplified by the disparities in COVID-19. Our health centers are adjusting. However, ask yourselves who is running with the EDIB initiatives, who are asked to educate everyone about equity, who is the expert, yet, not sitting at the highest levels of the organization.

## Conclusion

Academic medicine and healthcare systems need leaders that fundamentally value the wellness of their physician faculty and healthcare workforce; and in addressing wellness, we can also address equity and inclusion. What’s in it for organizations? Organizations that are inclusive foster employee well-being, employees with higher levels of well-being are shown to be more inclusive, and effective wellness initiatives can accommodate unique needs ( Menzies, 2018). Moreover, physicians of color care for 53.5% of diverse populations and 70.4% of non-English-speaking patients. Patients from underserved populations are significantly more likely to see a physician of color than white physicians ( Marrast *et al.,* 2014).It is our responsibility to address their wellness needs and that can only be done by addressing the complicit structural inequities within our academic and healthcare environments.

Bodenheimer and Sinsky reminded us that we need to expand to a quadruple aim: enhancing patient experience, improving population health, reducing costs, and improving the work-life of health care providers (2014). Shanafelt *et al.* demonstrated the impact of organizational leaders on the well-being and satisfaction of individual physicians (2015). As champions for equity and wellness, we assert that these are not exclusive from each other. May this article become a call to action for all wellness leaders to consider equity and inclusion as part of their own work. Lastly, we offer a simple reminder: We must have inclusive wellness conversations. Dialogue about wellness without equity ignores a simple truth -the wellness of our minoritized physicians and faculty cannot occur without equity.

## Take Home Messages

• Although the wellness literature encourages a review of organizational challenges, local needs, and individual solutions, organizations may fail to align these efforts along with equity, diversity, inclusion, and belonging (EDIB).
• Dialogue about wellness without equity ignores a simple truth -the wellness of our minoritized physicians and faculty cannot occur without equity.
• It is the onus of the organization to create a space where minoritized physicians feel safe to share their insights; and not the responsibility of the physician to try to carve out a niche in an organization that is not attuned to them.
• Based on seven aspects of wellness: career, financial, spiritual, physical, intellectual, family, and social needs, we pose questions for consideration as an example that can be adapted by institutions’ wellness leads.
• Leading with equity and wellness in mind should inform decision-making.

## Notes On Contributors

Sylk Sotto-Santiago, EdD, MBA, MPS, Vice-Chair for Faculty Affairs, Development and Diversity. Assistant Professor of Medicine, Department of Medicine, Indiana University School of Medicine. ORCiD: https://orcid.org/0000-0003-3810-1205

Dianne Ansari-Winn, MD, MPH, Founder, The Physician Coaching Academy, Denver, Colorado.

Chemen Neal, MD, Assistant Professor of Clinical Obstetrics and Gynecology, Department of Obstetrics and Gynecology, Indiana University School of Medicine.

Michael Ober, MD, MS, Vice-Chair for Clinical Affairs and Wellness, Associate Professor of Clinical Medicine, Department of Medicine, Indiana University School of Medicine.
