# Supplementary material for: Equity + Wellness: A Call for More Inclusive Physician Wellness Efforts
Source: MedEdPublish (2016). 2021 Apr 26;10:99. [Version 1] doi: 10.15694/mep.2021.000099.1 (PMC10939658; doi:10.15694/mep.2021.000099.1)
Supplement: Peer review history

# Reviewer report: Reviewer response for version 1

**DOI:** 10.21956/mep.19095.r27519

**Published:** 2021-10-09

**Authors:** Balakrishnan Nair

**Affiliations:** 1 Centre for Medical Professional Development and University of Newcastle

This review has been migrated. The reviewer awarded 4 stars out of 5 The wellness of the health workforce is vital if we have to provide health care and wellness to our patients. We need to think of the inclusiveness of all health care workers irrespective of their colour, race, religion and ethnicity. We live in a multicultural society and the workforce reflects this. Minorities and women have more stress and burnout in medicine. As the authors state, equity and wellness should go hand in hand. It is the duty of the majority to look after the minority and we have a long way to go as the authors state. So this review is very stimulating with some solid evidence to support each argument . So their call to keep equity and wellness as a top agenda item is very timely. A minor suggestion----"incentives carry a risk of bias toward women and underrepresented physicians” may be better stated as "bias against "

Reviewer Expertise:

NA

No decision status is available

# Reviewer report: Reviewer response for version 1

**DOI:** 10.21956/mep.19095.r27518

**Published:** 2021-10-08

**Authors:** Julie Hunt

**Affiliations:** 1 Lincoln Memorial University

This review has been migrated. The reviewer awarded 4 stars out of 5 In this paper, the authors make a timely, well justified opinion statement that wellness programs and initiatives fundamentally must include a consideration of diversity, equity, inclusion, and belonging. The authors assert that there can be no wellness if there is no equity, for example, which is a good point. The authors make several other points that would be beneficial to those designing or leading wellness initiatives: that focusing on personal resilience can shift some of the blame for un-wellness onto the individual, and that there should be simultaneous focus on institutional resilience for the sake of balance.Overall, this article may be of interest to those who design and implement wellness programs, and to the leadership at institutions who must make decisions that potentially impact the wellness of their employees.

Reviewer Expertise:

NA

No decision status is available

# Reviewer report: Reviewer response for version 1

**DOI:** 10.21956/mep.19095.r27517

**Published:** 2021-07-30

**Authors:** Anita Samuel

This review has been migrated. The reviewer awarded 4 stars out of 5 This is a very timely article that provides an overlooked aspect of wellness – the relationship between equity practices and wellness. The article is organized in an interesting format with the authors posing questions that institutions can use to guide their wellness + equity initiatives. While this article is framed in the context of the US, it has global relevance. Below are some suggestions to strengthen this article:• Including a reference for the Wheel of Life would be helpful. • In the Equity+Wellness section, Spirituality and Family are combined with the section on Physical. Was this intentional? They might work better if they were separated into another paragraph. • There are a few odd sentence structures that should be edited for clarity. For example, the third paragraph in Introduction is an incomplete sentence. • In the Conclusion you state “in addressing wellness, we can also address equity and inclusion.” Shouldn’t this be the other way round? In addressing equity, we also address wellness.• In the Career section, you use the acronym EDIJ. I think it should be EDIB.

Reviewer Expertise:

NA

No decision status is available
